# Repair of Osteochondral Defects Using Human Umbilical Cord Wharton's Jelly-Derived Mesenchymal Stem Cells in a Rabbit Model

**DOI:** 10.1155/2017/8760383

**Published:** 2017-02-05

**Authors:** Shuyun Liu, Yanhui Jia, Mei Yuan, Weimin Guo, Jingxiang Huang, Bin Zhao, Jiang Peng, Wenjing Xu, Shibi Lu, Quanyi Guo

**Affiliations:** ^1^Beijing Key Laboratory of Regenerative Medicine in Orthopaedics, Key Laboratory of Musculoskeletal Trauma & War Injuries, PLA, Institute of Orthopaedics, Chinese PLA General Hospital, Beijing 100853, China; ^2^Department of Orthopaedics, The 251st Hospital of Chinese PLA, Zhangjiakou 075000, China

## Abstract

Umbilical cord Wharton's jelly-derived mesenchymal stem cell (WJMSC) is a new-found mesenchymal stem cell in recent years with multiple lineage potential. Due to its abundant resources, no damage procurement, and lower immunogenicity than other adult MSCs, WJMSC promises to be a good xenogenous cell candidate for tissue engineering. This in vivo pilot study explored the use of human umbilical cord Wharton's jelly mesenchymal stem cells (hWJMSCs) containing a tissue engineering construct xenotransplant in rabbits to repair full-thickness cartilage defects in the femoral patellar groove. We observed orderly spatial-temporal remodeling of hWJMSCs into cartilage tissues during repair over 16 months, with characteristic architectural features, including a hyaline-like neocartilage layer with good surface regularity, complete integration with adjacent host cartilage, and regenerated subchondral bone. No immune rejection was detected when xenograft hWJMSCs were implanted into rabbit cartilage defects. The repair results using hWJMSCs were superior to those of chondrogenically induced hWJMSCs after assessing gross appearance and histological grading scores. These preliminary results suggest that using novel undifferentiated hWJMSCs as seed cells might be a better approach than using transforming growth factor-*β*-induced differentiated hWJMSCs for in vivo tissue engineering treatment of cartilage defects. hWJMSC allografts may be promising for clinical applications.

## 1. Background

Damage to articular cartilage is usually caused by sports injuries, accidental trauma, and aging. After a traumatic or pathological injury, hyaline articular cartilage, which is the load-bearing joint tissue, has very limited or no intrinsic capacity for self-repair, and even minor lesions or injuries can lead to progressive damage and joint degeneration. Frequently used treatments for articular cartilage damage, such as surgical interventions (microfracture and osteochondral auto- or allografts), are less than satisfactory, rarely restore full function, and may lead to fibrocartilage but not hyaline articular cartilage in the long-term. Repair and regeneration of cartilage remains a challenge in orthopedic surgery. The long-term success of cartilage repair depends on regenerative methodologies that restore articular cartilage to a close duplicate of native tissue.

The development of tissue engineering-based cartilage repair methods has been pursued to provide more functional biological tissues. Autologous chondrocyte implantation (ACI) based engineered cartilage was first reported by Brittberg et al. in 1994 [1]; however, this treatment requires extracting chondrocytes directly from the patient, thus inducing additional donor site morbidity of healthy articular cartilage.

Mesenchymal stem cells (MSCs) are a rare population of multipotent precursors that can be isolated from many different tissues and differentiate into different lineages under appropriate conditions. Furthermore, MSCs are an attractive cell source for therapeutic applications due to their potential to secrete trophic and immunomodulatory molecules [2]. Multipotent adult MSCs can also differentiate into cells of the chondrogenic lineage [3], which has led to a variety of experimental strategies to investigate whether MSCs can take the place of chondrocytes during regeneration and maintenance of articular cartilage. Plus, MSCs are readily available in large quantities, easy to isolate without significant donor site morbidity, and can be expanded easier in vitro compared with chondrocytes. MSCs can also synthesize an ECM with properties that closely mimic healthy hyaline joint cartilage [4]. In addition, these cells may also influence the course of chronic degenerative disorders and prevent cartilage degradation in patients with osteoarthritis (OA) through their trophic/regenerative potential. Preclinical studies performed using MSCs and predifferentiated cartilage cells have shown promising results.

Although human BM-MSCs have been studied extensively and are widely used, harvesting of these cells is highly invasive, and the frequency, proliferation efficiency, and differentiation potential of BM-MSCs decline with age [5]. Another group of MSCs has been identified in virtually all postnatal organs and tissues [6] and could represent an alternative source of adult MSCs. Fetal or neonatal MSCs appear to be more primitive and have greater multipotentiality than their adult counterparts. Several studies have reported superior cell biological properties, such as improved proliferative capacity, lifespan, and differentiation potential of MSCs from birth-associated tissues compared to BM-MSCs. Their intermediate state between adult and embryonic stem cells (ESCs) also makes them an ideal candidate for reprogramming to pluripotent status. Fetal MSCs are very attractive for a wide range of regenerative medicine applications [7]. MSCs in Wharton's jelly (WJ) from the umbilical cord (UC) have desirable characteristics. First, the UC, which is discarded at birth, provides an inexhaustible source of stem cells for therapy. In addition, Wharton's jelly-derived MSCs (WJMSCs) from the UC have faster proliferation rates and greater expansion capability than those of adult MSCs, and they possess broad multipotency and do not induce teratomas [8, 9]. WJMSCs are believed to be more primitive than MSCs derived from other tissue sources and express both MSC and ESC markers [10]. What is more, the WJ collection procedure is noninvasive and painless, and WJMSCs are an ethically noncontroversial source of MSCs. Thus, human WJMSCs could be prospectively used in cartilage tissue engineering and cell-based therapy for OA, due to their abundant supply, easy procurement, robust proliferation, and high purity compared with BM-MSCs and MSCs from adipose tissue. In addition, the composition of Wharton's jelly extracellular matrix is very similar to that of cartilage extracellular matrix, and the hWJMSCs express aggrecan, type II collagen, and SOX-9 as chondrocytes do [11]. hWJMSCs also express cell growth factors, chemokines, and cytokines at levels similar to those of cartilage cells [12].

However, using hWJMSCs as allogeneic donor cells inevitably raises the question as to whether they are immunogenic, and if so, would they be rejected after transplantation. Thus, the immunogenicity of hWJMSCs was an initial concern. Two outstanding features of MSCs are relevant to immunity: (1) immunosuppression, through specific interactions with immune cells that participate in the innate and adaptive responses, and (2) so-called immunoprivilege, the mechanisms of which are most probably due to low expression levels of major histocompatibility complex- (MHC-) I, MHC-II, and costimulating factors. Based on their immunosuppressive functions and immunoprivilege,* MSCs* do not challenge the response of allogeneic immune cells. The immunosuppressive effects of BM-MSCs have been studied extensively [13], but neonatal and adult MSCs exhibit considerable differences in their functional abilities. The immune characteristics of neonatal MSCs have been less reported compared with those of adult BM-MSCs [14]. Based on our previous study, hWJMSCs possess hypoimmunogenic properties in vitro and in vivo. hWJMSC immunoprivilege was identified because they express HLA-ABC (MHC-I) in a subpopulation of approximately 76%, while most cells (>99.7%) do not express HLA-DPDQDR (MHC-II). Also, hWJMSCs do not express costimulators, such as CD40, CD80, or CD86, which are also necessary to activate T cells [15]. The immunosuppressive properties of hWJMSCs have been characterized by their capacity to inhibit proliferation and function of all immune cells [16]. Weiss et al. [17] showed that hWJMSCs inhibit the splenocyte response to Concanavalin A stimulation in vitro and do not stimulate T-cell proliferation in a one-way mixed lymphocyte reaction (MLR) assay but inhibit proliferation of stimulated T cells in a two-way MLR assay. Immunomodulation in hWJMSCs is induced principally through soluble mediators, and indoleamine 2, 3-dioxygenase is a major player among them [18]. Besides, transforming growth factor- (TGF-) *β*1, hepatocyte growth factor, heme oxygenase 1, interleukin- (IL-) 6, leukemia inhibitory factor, human leukocyte antigen G, IL-10, IL-1 receptor antagonist, and prostaglandin E2 have been proposed as other mediators involved in hWJMSC-mediated immunomodulation [14, 17]. Subcutaneously transplanting hWJMSCs into rats and rabbits results in limited infiltration of CD4+ and CD8+ T cells, and hWJMSCs remained alive for 4 weeks after implantation, revealing only weak or no immune rejection [15]. Weiss et al. also implanted hWJMSCs into rat brains and reported the same results [19]. Differences between hBMMSCs and hWJSCs in terms of immunogenicity have been demonstrated. A mild lymphoproliferative response to BM-MSCs but not hWJSCs was reported when these two cell types were cocultured with human peripheral blood mononuclear cells [14]. Taken together, these results suggest that using WJMSCs for allograft therapy may not or may only weakly induce immune rejection.

These results suggest that WJMSCs are a prospective cell candidate for cartilage tissue engineering; however, experimental allogeneic transplantation of human WJMSCs cannot be performed. Thus, in this study, we examined inflammation of hWJMSCs in nude rats and evaluated their effects using xenotransplantation to repair cartilage defects in rabbits.

## 2. Methods

### 2.1. Animal

4-week-old nude rats (Crl:NIH-Foxn1rnu; weight, 200–260 g) were purchased from Beijing WeiTongLiHua Co. Male New Zealand white rabbits were purchased from the Laboratory Animal Research Center of General Hospital. Animal experiments were performed in accordance with the Institutional Animal Care and Use Committee of General Hospital.

### 2.2. Materials and Reagents

Dulbecco's Modified Eagle Medium (DMEM) and F12 medium (Sigma, St. Louis, MO, USA), fetal bovine serum (FBS) (Beijing Yuanheng Jinma, China), TGF-*β*1 (PEPRO Tech, Hamburg, Germany), and fibroblast growth factor (FGF) (PEPRO Tech) were purchased. Agents for histochemical staining, including toluidine blue (Beijing Chemical Reagent Co. Beijing, China), antibodies for flow cytometry (BD Pharmingen, San Diego, CA, USA), PE-conjugated mouse anti-human antibody (BD Pharmingen), and human ribonucleoprotein antibody (Chemicon, Temecula, CA, USA), were purchased.

### 2.3. hWJMSC Isolation and Culture

Human UCs were collected aseptically from the maternity department at General PLA Hospital from normal full-term births with informed consent. The umbilical arteries and vein were removed, and the remaining tissue was diced into small fragments. Explants were transferred to culture flasks containing DMEM/F-12 and 10% FBS. They were left undisturbed for 5–7 days to allow the cells to migrate from the explants, and then the media were replaced. Cells were refed and passaged as necessary.

### 2.4. Flow Cytometry

Passage-4 hWJMSCs were selected for flow cytometry analysis. Cells were trypsinized and suspended in phosphate-buffered saline (PBS, pH 7.4) at a concentration of 5 × 10^6^/ml, and a 100 *μ*l sample was incubated with various FITC/PE-labeled mouse anti-human antibodies for 45 min at room temperature. Cells were washed twice with PBS, centrifuged, and resuspended in 0.5 ml PBS. Control samples were incubated with PBS instead of antibody. A FACScan machine (Gilson, Villiers-le-Bel, France) was used to analyze antibody binding.

### 2.5. Cell-Scaffold Construction

Scaffolds derived from the extracellular matrix (ECM) of swine cartilage, 4 mm in diameter and 1.5 mm high, were prepared as described previously [20, 21] and sterilized with Co_60_ irradiation. Passage-4 hWJMSCs (3 × 10^7^ cells/ml) were injected into each scaffold and the cell-scaffold constructs were cultured in normal culture medium (DMEM/F-12, 10% FBS) for hWJMSCs-ECM group and in chondrogenic induction medium (containing TGF-*β*1 (10 ng/ml), FGF (25 ng/ml), ITS (1 : 100), and dexamethasone (10^−7^ M), without FBS) for hWJMSC-Cs-ECM group. Both groups were cultured for 14 days.

### 2.6. Cell Viability Assay

Cell viability was measured in the three-dimensional scaffold cultures (hWJMSCs or chondrogenically induced hWJMSCs [hWJMSC-Cs]). The constructs were rinsed in PBS and stained with FDA (5 *µ*g/ml) for 5 min, followed by a 5 min PBS rinse and staining with propidium iodide (PI) (5 *μ*g/ml). Cell viability was determined under a confocal microscope (Leica TCS-SP8) and quantified with Image-pro Plus software.

### 2.7. Scanning Electron Microscopy (SEM)

Cell-seeded scaffolds were analyzed by SEM (S-2600N; Hitachi Science Systems, Ltd., Tokyo, Japan) for further characterization of hWJMSC growth. SEM was performed with fixed constructs. After dehydrating the samples in a graded ethanol series, the samples were dried in a critical point dryer and coated with gold-palladium (EMS850X; Electron Microscopy Sciences, Hatfield, PA, USA).

### 2.8. Cartilage Defect Rabbit Model

Under general anaesthesia, the rabbits' knee joints were opened with a medial parapatellar approach. A full-thickness cylindrical defect (4 mm in diameter, 1.5 mm deep) was created on the patellar groove of the femur in both legs, using a corneal trephine. The rabbit defects were divided into four groups: (a) untreated group, (b) cartilage ECM-derived scaffolds alone group, (c) scaffolds with undifferentiated hWJMSCs group (hWJMSCs-ECM), and (d) scaffolds with chondrogenically differentiated hWJMSC-Cs group (hWJMSC-Cs-ECM). Specimens were harvested 3, 6, 12, and 16 months postoperatively, followed by gross observations, histological analysis, and GAG content measurements. Gross morphology was evaluated according to the International Cartilage Repair Society (ICRS) score [22]. The ICRS score consists of four categories (degree of repair, integration, surface regularity, and total judgment) and is scored as 0–12 points, in which 12 represents complete regeneration and 0 is no regeneration.

### 2.9. Histology Staining and Scoring

Samples for histological analysis were fixed in 10% neutral-buffered formalin, decalcified in 10% EDTA, and embedded in paraffin. Sections were cut to 5 *μ*m thickness, deparaffinized, and stained with hematoxylin and eosin (H&E) and toluidine blue (TB) following standard procedures described previously. Human-specific ribonucleoprotein immunohistochemical staining was carried out to detect implanted hWJMSCs. Briefly, the sections were deparaffinized, rehydrated, and then treated with 3% v/v hydrogen peroxide to block endogenous peroxidase activity, followed by digestion with pepsin solution at 37°C for 15 min. The sections were subsequently incubated with 10% horse serum in PBS v/v at room temperature for 30 min, to reduce nonspecific staining before overnight incubation with primary antibody at 1 : 100 dilution in PBS containing 0.1% w/v BSA at 4°C. The sections were then incubated with horseradish peroxidase- (HRP-) conjugated secondary antibody at 1 : 500 dilution for 30 min at room temperature. Finally, the sections were colorated with DAB, mounted in resin, and viewed by optical microscopy. Sections incubated with PBS without primary antibodies were used as a negative control.

Histological sections of the repaired tissue were analyzed in a blind manner by two observers who were not informed of the group assignments, and sections were quantified using the histological grading system for cartilage defects described by the ICRS-I score [23]. This system consists of 6 categories (surface, matrix, cell distribution, cell population viability, subchondral bone, and cartilage mineralization) and is scored as 0–18 points, in which 18 represents complete regeneration and 0 is no regeneration.

### 2.10. Tissue GAG Levels

Tissues sampled 6 months after transplantation were subjected to a GAG assay. Osteochondral plugs within the repair area were cored out using a 4 mm internal diameter core bit fixed to a standing drill press. Cartilage slices (2 mm thick) were cut for biochemical analysis and same-thickness discs from the surrounding cartilage were harvested as controls. The samples were freeze-dried and digested in 1 ml papainase (1.25 mg/ml Papain, 100 mM Na_2_HPO_4_, 10 mM EDTA, and 10 mM cysteine, pH 6.3) for 18 h at 60°C. GAG content was evaluated by dimethylmethylene blue (Sigma) staining of chondroitin sulfate, as reported by Farndale et al. (1986).

### 2.11. Subcutaneous Implantation of Cell-Scaffold Constructs in Nude Rats

Scaffolds derived from ECM of pig cartilage were used for cell-scaffold construction and subcutaneous implantation in nude rats, and our experiment demonstrated that they did not cause immune response when implanted into rabbits [24]. The hWJMSCs-ECM or hWJMSC-Cs-ECM constructs were implanted subcutaneously into the backs of nude rats, and cell inflammation was investigated by histochemical staining after the observation period.

### 2.12. Statistical Analysis

All quantified results are expressed as the mean ± standard deviation (SD). One-way analysis of variance (ANOVA) followed by Bonferroni's multiple comparison test was used to detect significant differences between groups. A *p* value < 0.05 was considered significant.

## 3. Results

### 3.1. Characterization and Surface Markers for Cells Derived from hWJ

**Figure 1:**
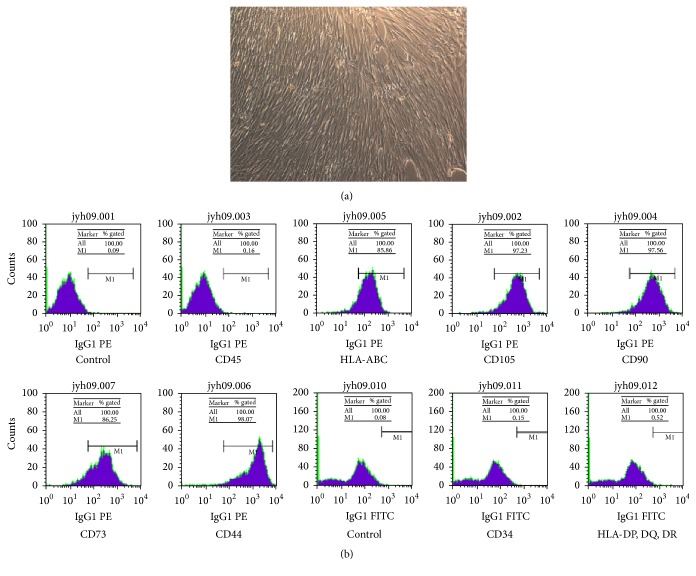
Characteristics of human umbilical cord Wharton's jelly mesenchymal stem cells (hWJMSCs). (a) Morphology of cultured primary hWJMSCs, in which the cells had fibroblast-like shapes and were a homogeneous cell population. (b) Flow cytometric analysis of surface marker expression on hWJMSCs, in which CD44, CD90, CD105, CD166, CD73, and HLA-ABC were positive and CD34, CD45, and HLA-DPDQDR were negative.

hWJMSCs began to migrate from the explants after 5–7 days in culture with DMEM/F-12 and 10% FBS.  Figure 1 shows a phase-contrast view of the spindle-shaped adherent hWJMSCs under an optical microscope. Flow cytometry revealed that the isolated cells were positive for MSC markers, including CD44, CD73, CD90, CD105, and HLA-ABC, but negative for endothelial and hematopoietic markers, including CD34, CD45, and HLA-DPDQDR (Figure 1(b)).

### 3.2. Cartilage Regeneration in Joint Articular Defects of Rabbits

**Figure 2:**
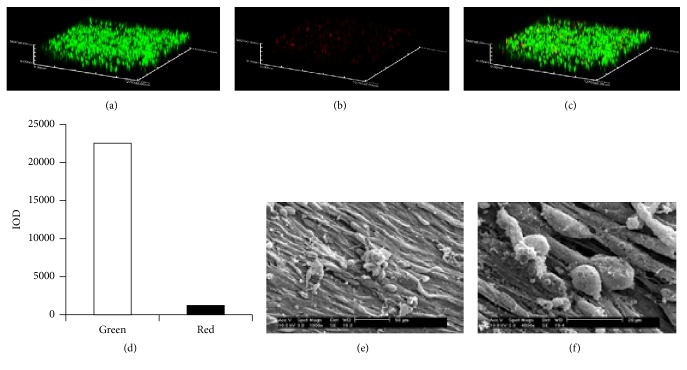
Human umbilical cord Wharton's jelly mesenchymal stem cells (hWJMSCs) cultured on cartilage extracellular matrix-derived scaffolds. Most cells were alive when stained with fluorescein diacetate (FDA) (a) and propidium iodide (PI) (b), (c) merge, (d) quantification of the live and dead cells in the confocal image. Scanning electron microscopic (SEM) examination revealed that cells grew well on the scaffolds after 14 days of culture ((e) and (f)).

Both the hWJMSCs and hWJMSC-Cs attached to the scaffolds and proliferated for up to 2 weeks. Cell viability assay showed that most cells were alive when stained with fluorescein diacetate (FDA) and propidium iodide (PI), and quantification of the live and dead cells in the confocal image also showed the same results (Figure 2). Then we used the hWJMSCs/ECM and hWJMSC-Cs/ECM constructs to repair rabbit cartilage defects and assessed cartilage regenerative capability of hWJMSCs after transplantation into a critical-sized cartilage defect by means of a gross morphology examination, histological and immunohistochemical evaluations, and a semiquantitative GAG analysis.

### 3.3. Gross Appearance

**Figure 3:**
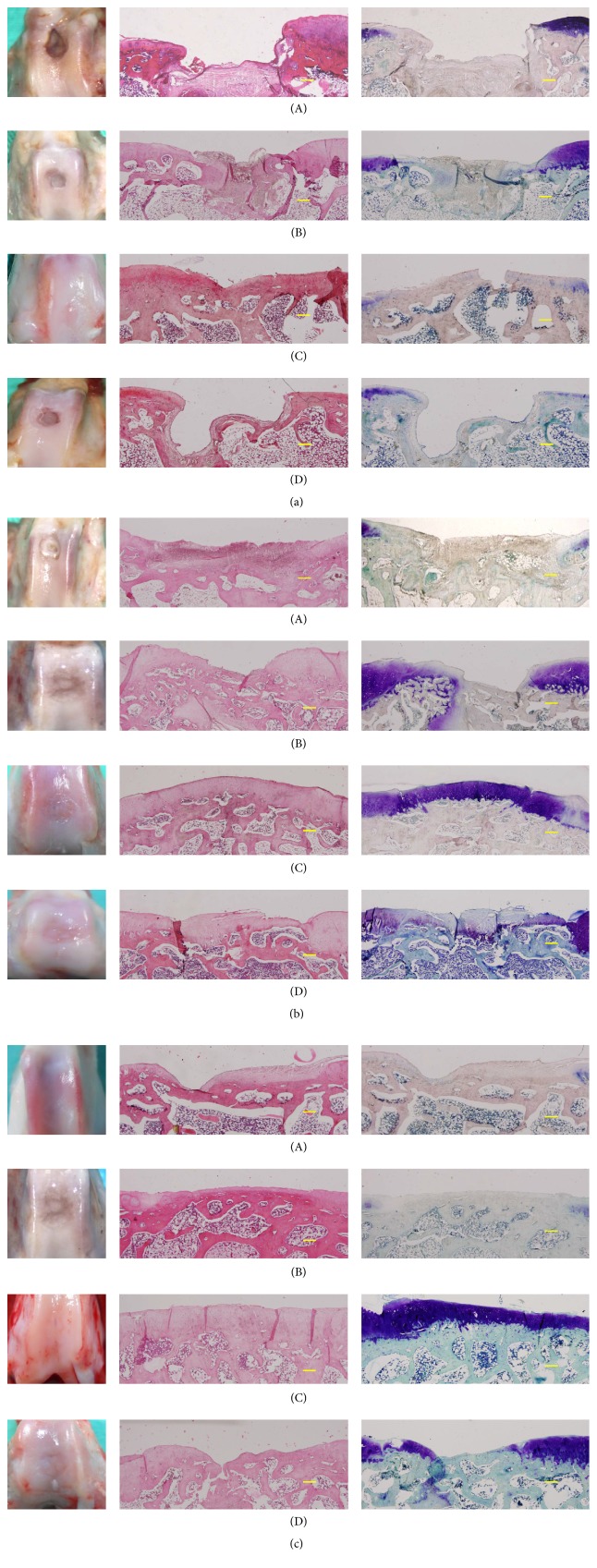
Total gross appearance and histological results of representative samples. (a) 3 months' group, (b) 6 months' group, and (c) 12 months' group. The histological analysis included hematoxylin and eosin (H&E) and toluidine blue staining. Animals were randomly assigned to four groups: (A) untreated group, (B) extracellular matrix (ECM) scaffold alone group, (C) ECM scaffold loaded with human umbilical cord Wharton's jelly mesenchymal stem cells (hWJMSCs) group, and (D) ECM scaffold loaded with hWJMSC-Cs group (hWJMSC-Cs-ECM). Only empty defects but no repaired tissues were detected in the untreated and scaffold alone groups 3 months after implantation. The defects were partially repaired with hyaline cartilage in the hWJMSCs-ECM group, whereas most defects were filled with fibrous tissue in the hWJMSC-Cs-ECM group. Partially repaired fibrosis tissue was found in the untreated and scaffold alone groups 6 months after implantation. Partial fibrocartilage or hyaline cartilage was found in the hWJMSCs-ECM and hWJMSC-Cs-ECM groups, and hyaline cartilage was more abundant in the hWJMSCs-ECM group than in the hWJMSC-Cs-ECM group. Hyaline cartilage had completely bonded to adjacent cartilage, with restored and fully resurfaced subchondral bone in the hWJMSCs-ECM group 12 months after implantation. The concave open boxes became smaller than before and were repaired with hyaline cartilage in the hWJMSC-Cs-ECM group. However, all defects in the untreated and scaffold groups were repaired with fibrosis tissue. Bar: 200 *μ*m.

**Figure 4:**
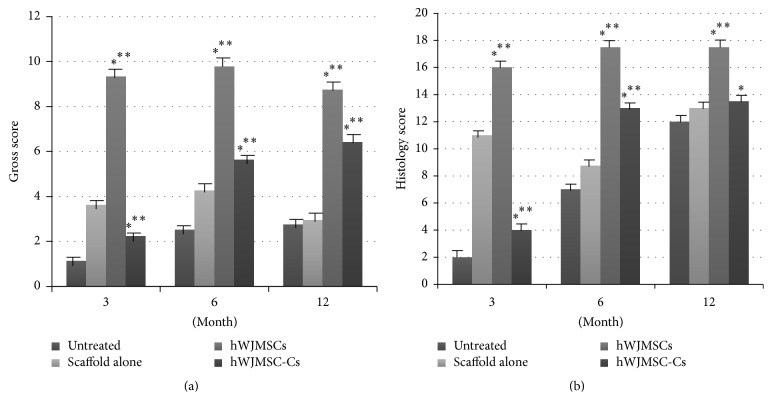
Gross and histological scores for repaired tissues in rabbit cartilage defects. (a) Gross score, the hWJMSCs-ECM and hWJMSC-Cs-ECM groups were significantly different (*p* < 0.05^*∗*^) 3, 6, and 12 months after transplantation. Both the hWJMSCs-ECM and hWJMSC-Cs-ECM groups were significantly different from the untreated and scaffold alone groups (*p* < 0.05^*∗∗*^). (b) ICRS histological score, the hWJMSCs-ECM and hWJMSC-Cs-ECM groups were significantly different (*p* < 0.05^*∗*^) 3, 6, and 12 months after transplantation. Both the hWJMSCs-ECM and hWJMSC-Cs-ECM groups were significantly different from the untreated and scaffold alone groups (*p* < 0.05^*∗∗*^), except the hWJMSC-Cs-ECM group at 12 months.

3 months postoperatively, all defects in the untreated and scaffold alone groups appeared as contracted craters and were depressed and irregular with poor integration with the host cartilage (poor). In contrast, defects in the hWJMSCs-ECM group consisted of a single smooth fully repaired surface (good), four partially repaired surfaces (fair), and one poorly repaired surface (poor). Two defects in the hWJMSC-Cs-ECM group had partially repaired surfaces (fair) and three had poorly repaired surfaces (poor). At 6 months, one poor and two fair results were observed in the untreated group; two poor and one fair results in the scaffold alone group; one poor, two fair, and three good results in the hWJMSCs-ECM group; and one poor and five fair results in the induced hWJMSC-Cs-ECM group. At 12–16 months, all defects were poor, except one fair in the untreated and scaffold alone groups; three defects showed good surfaces and one was fair in the hWJMSCs-ECM group; one poor, one fair, and one good result were detected in the hWJMSC-Cs-ECM group. All results and gross scores are shown in Figures 3 and 4(a) and Table 1.

### 3.4. Histological Results

**Figure 5:**
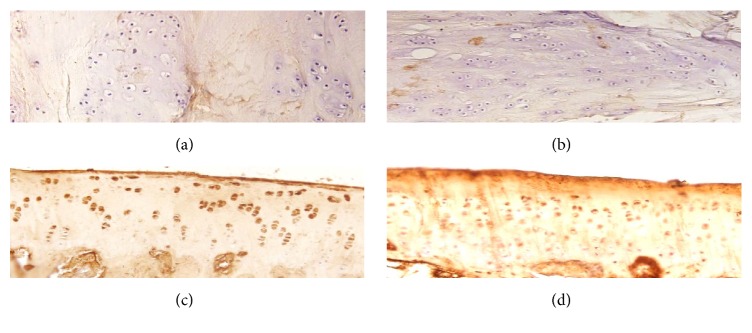
Identification of implanted cells by anti-human nuclear protein immunohistological staining. The staining results were negative in groups (a) and (b) and positive in groups (c) and (d) at 12 months after transplantation.

At 3 months, defects in the untreated and scaffold alone groups were uneven and filled with fibrous tissue. In the hWJMSCs-ECM group, the defect areas were filled with hyaline-like cartilage mixed with some fibrocartilage, and subchondral bone had formed completely. Defect areas in the hWJMSC-Cs-ECM group were filled with fibrous tissue in most cases and central hyaline-like cartilage in two rabbits (Figure 3(a)). At 6 months, most of the hWJMSCs- and hWJMSC-Cs-treated defects had a moderately flat regular surfaced neotissue graft. The TB staining results revealed similar GAG production in the hWJMSC-Cs-ECM and hWJMSCs-ECM groups, and both groups produced more GAG than the untreated and scaffold alone groups. More subchondral bone was evident in the hWJMSCs-ECM group than in the hWJMSC-Cs-ECM group as TB staining showed. Additionally, the repaired tissues were stained with human ribonucleoprotein antibody to detect the implanted human hWJMSCs-ECM, and both hWJMSCs-ECM and hWJMSC-Cs-ECM groups were positive, demonstrating that the implanted cells were alive. In contrast, the untreated and scaffold alone groups displayed moderate fibrous tissue coverage, and the human ribonucleoprotein staining results were negative (Figure 3(b)). At 12–16 months, the hWJMSCs-ECM-treated defects displayed good surface regularity with abundant cartilage matrix, significant integration of newly formed tissue with surrounding normal cartilage, and appeared similar to the native control, but a significant difference in histological grading score was found compared with the native control group. Only one defect in the hWJMSC-Cs-ECM treated group displayed good surface regularity and hyaline-like cartilage, whereas the other two defects were repaired with fibrous tissue or fibrocartilage cells (Figures 3(c) and 5).

Combining the gross appearance and histological results, the hWJMSCs-ECM group yielded higher ICRS gross and histology scores than those in the hWJMSC-Cs-ECM group. The highest score in the hWJMSC-Cs-ECM group was achieved 6 months after implantation, with little decrease at 12–16 months after implantation. Histological results and scores are shown in Figures 3 and 4(b) and Table 2.

### 3.5. Quantitative GAG Content Analysis 

GAG content in cartilage samples was 28.42 ± 0.17 *μ*g in the hWJMSCs-ECM group and 7.48 ± 0.26 *μ*g in the hWJMSC-Cs-ECM group 6 months after implantation, both of which were lower than that of healthy cartilage (36.22 ± 1.51 *μ*g). The mean ± SD GAG level was higher in the hWJMSCs-ECM group than in the hWJMSC-Cs-ECM group (*p* < 0.05).

### 3.6. Subcutaneous Implantation into Nude Rats 

**Figure 6:**
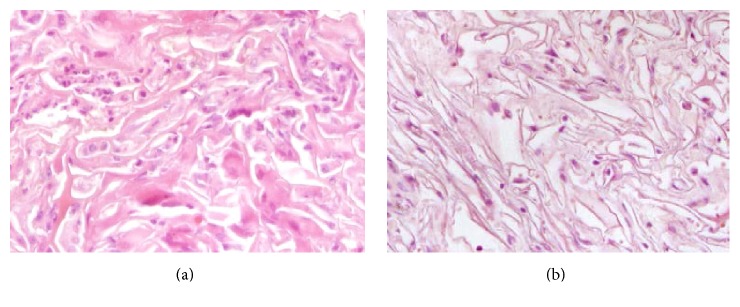
Inflammatory reactions in hWJMSC-Cs/ECM and hWJMSCs/ECM constructs implanted in nude rats. Granular leucocytes and macrophages infiltrated around the hWJMSC-Cs/ECM implants 18 days after implantation (a), whereas no inflammatory infiltration was observed in the hWJMSCs/ECM implants (b).

The cell-scaffold constructs were removed 18 days after implantation, and a histochemical analysis was performed. H&E staining results showed that granular leucocytes and macrophages had infiltrated around the hWJMSC-Cs/ECM implants, but no inflammatory infiltration was detected in the hWJMSCs/ECM implants (Figure 6), probably due to the multiple growth factors and cytokines hWJMSCs secreted with anti-inflammatory and apoptosis effect.

## 4. Discussion

Cartilage defects have been repaired with BM-MSC or adipose tissue-derived MSC constructs in a number of recent studies with good outcomes. However, no study has applied hWJMSCs for cartilage repair in an orthotopic model. This in vivo pilot study explored the use of hWJMSC-containing tissue engineering xenografts to repair cartilage defects created in the knees of rabbits.

### 4.1. Were the Xenografts Rejected?

We previously examined whether hWJMSC xenografts transplanted into rabbits were immune rejected. The results showed no T lymphocyte infiltration around the implanted cells at the early stage (1–4 weeks after hWJMSCs and hWJMSC-Cs implantation). The hWJMSCs remained alive and were identified by human ribonucleoprotein immunostaining. In this study, the hWJMSCs and hWJMSC-Cs implanted into the articular cartilage defects did not induce immune rejection and were alive 12–16 months after transplantation. This result reveals the hypoimmunogenic properties of hWJMSCs, which agrees with some recent reports. Pig UC-MSCs injected into the brains of Parkinsonian rats were not rejected, survived, and proliferated for up to 4 weeks, producing tyrosine hydroxylase-positive neurons that expressed porcine-specific markers [25], as shown in human ESC-derived cartilage cells implanted in rats [26] and human BMSC-derived cartilage cells transplanted into rabbits [27–29]. Fan et al. [30] reviewed several studies that preclinically validated UC-MSCs or derived tissues in diseased animal models. In all of the studies, the UC-MSCs differentiated and engrafted successfully in rat models of cerebral ischemia, intracerebral hemorrhage, spinal cord injury, Parkinson's disease, retinal disease, type 1 diabetes, and myogenic disease. Li et al. reviewed studies through 2011 and identified 94 reports on in vivo cross-species administration of MSCs in a variety of experimental models. The majority (*n* = 89) involved use of human MSCs in various species, and the human MSCs were derived mainly from BM, adipose tissue, or UC blood. Results from 88 experiments (93.6%) showed that the MSCs engrafted and functioned across species barriers, and failure to function was reported in only six cases (6.4%) [31]. In addition, MSCs can prolong graft survival and induce tolerance in some cases. Applying MSCs to solid organ transplantation is now being evaluated in phase I/II clinical trials [32].

### 4.2. hWJMSCs Differentiated into Cartilage Cells In Vivo

Animal model results indicate that BM-MSCs or adipose-derived stem cells without preinduction can be used to repair cartilage defects [33–36]. Milano et al. reported that cartilage ECM with platelet-rich plasma scaffolds combined with microfractures to treat chondral defects improve mechanical and biochemical quality of repaired tissue because the stem cells from bone marrow move to the cartilage defects and differentiate into cartilage cells in the cartilage niche in vivo [37]. In this study, we transplanted the hWJMSCs into cartilage defects of rabbits and determined that hWJMSCs differentiated into cartilage cells and repaired cartilage defects.

### 4.3. Should MSCs Be Induced to Undergo Chondrogenesis to Treat Cartilage Defects?

In this preliminary pilot study, we found that hWJMSCs produced superior healing results to those of induced hWJMSCs, particularly during the early phase. This may have occurred because MSCs secrete a variety of cytokines and growth factors with paracrine and autocrine activities. These secreted factors suppress the local immune system, inhibit fibrosis (scar formation) and apoptosis, and stimulate mitosis and differentiation of tissue-intrinsic repair or stem cells [38, 39]. Wu showed that stimulating chondrocyte proliferation and matrix deposition increases cartilage formation, mainly due to the trophic role of MSCs rather than the MSCs actively undergoing chondrogenic differentiation [39]. In our study, granular leucocytes and macrophages infiltrated around the hWJMSC-C-ECM implants when hWJMSC-ECM and hWJMSC-C-ECM constructs were implanted in nude rats, whereas no inflammatory infiltration occurred around the hWJMSC-ECM implants. This result suggests that inflammation may delay cartilage repair. The ability of MSCs to produce paracrine factors with anti-inflammatory properties has been reported [40–43], and human UC-MSCs are useful to treat inflammatory arthritis [44]. This may explain, at least in part, why hWJMSCs have better repair effects than those of hWJMSC-Cs. But the inflammation type, also with the orthotopic site evaluation of the inflammatory reaction to the implant in the rabbits articular cartilage, remained unclear and needs further studies.

In addition, we found that the subchondral bone morphology in the repaired area was superior in the hWJMSCs-ECM group than in the hWJMSC-Cs-ECM group. We suppose that a fraction of the hWJMSCs retained stem cell characteristics in the undifferentiated implants and that this fraction was responsible for better restoration of the osteochondral junction. Karp and Leng Teo also reported that exogenous hWJMSCs have better homing and differentiating abilities than those of differentiated MSCs [45]. In contrast, TGF-*β*-pretreated hWJMSCs may be committed to chondrogenesis and have less ability to regenerate subchondral bone than undifferentiated cells. This result is similar to that of Chang et al., in which undifferentiated MSCs were better at repairing osteochondral defects in pigs than differentiated MSCs [46]. However, further studies are needed to substantiate this hypothesis.

Other studies have shown that differentiated MSCs are better than undifferentiated MSCs or the same [47, 48]. In our study, a slightly superior histological appearance of hyaline cartilage was detected in hWJMSCs than in hWJMSC-Cs after 12–16 months, whereas some studies demonstrated that the histological appearance of differentiated MSCs is better than that of undifferentiated MSCs after 1 year [49]. Also, there are some studies reporting that MSC grafts improve the early healing response but do not significantly enhance long-term histological appearance or biochemical composition of full-thickness cartilage lesions in sheep and equine models [49, 50]. Thus, the effectiveness of hWJMSC and hWJMSC-C repair activities remains controversial. These different results illustrate the need for more studies to determine the optimum conditions for producing tissue engineering constructs for cartilage repair.

### 4.4. How Long Can Human MSCs Remain Alive in Animal Models?

In our study, hWJMSCs were detectable in rabbit cartilage defect repair tissues 12 and 16 months after transplantation, whereas the number of human ESC-derived cartilage cells transplanted into rats decreased gradually after transplantation [26]. More studies are needed to determine how long human MSCs remain alive in animal models. Factors related to the fate of xenograft WJMSCs and ESCs and whether there are differences between them require more studies.

## 5. Conclusions

This experimental study demonstrated suitable hWJMSCs therapeutic conditions for repairing cartilage defects and should advance clinical application of hWJMSCs-based cell therapy for cartilage regeneration. Our results suggest that undifferentiated hWJMSCs may represent a better approach than TGF-*β*-induced differentiated hWJMSCs for in vivo tissue engineering treatment of cartilage defects. Further studies are needed to define the physiological mechanisms involved in hWJMSCs repair activities.

## Figures and Tables

**Table 1:** Total gross appearance of rabbit cartilage defects.

	No treatment	Scaffold alone	hWJMSC	hWJMSC-C
Contracted craters	7	7	2	5
Partially repaired surfaces	2	2	7	8
Smooth, fully repaired surfaces			7	1

**Table 2:** Histological analysis of repaired cartilage defects in rabbits.

	No treatment	Scaffold alone	hWJMSC	hWJMSC-C
Contracted craters	4	4	1	4
Fibrous tissue	4	5	2	5
Thin hyaline tissue	1		11	5
Total hyaline tissue			1	

## Abbreviations

hWJMSC: Human umbilical cord Wharton's jelly-derived mesenchymal stem cell
hWJMSC-C: Chondrogenically induced hWJMSCs
UC: Umbilical cord
ECM: Extracellular matrix
ACI: Autologous chondrocyte implantation
OA: Osteoarthritis
ESC: Embryonic stem cells
GAG: Glycosaminoglycans
MHC-I/II: Major histocompatibility complex-I/II
HLA-ABC/DPDQDR: Human leukocyte antigen
MLR: Mixed lymphocyte reaction
TGF-*β*1: Transforming growth factor
IL: Interleukin
DMEM: Dulbecco's Modified Eagle Medium
FBS: Fetal bovine serum
FGF: Fibroblast growth factor
PE: Phycoerythrin
PBS: Phosphate-buffered saline
FITC: Fluorescein isothiocyanate
SEM: Scanning electron microscopy
ICRS: International Cartilage Repair Society
MODS score: Modified O'Driscoll (MODS) score
EDTA: Ethylenediaminetetraacetic acid
H&E: Hematoxylin and eosin
TB: Toluidine blue
FDA/PI: Fluorescein diacetate/propidium iodide
BM-MSC: Bone marrow-derived MSC.
